# Polysaccharide Specific Monoclonal Antibodies Provide Passive Protection against Intranasal Challenge with *Burkholderia pseudomallei*

**DOI:** 10.1371/journal.pone.0035386

**Published:** 2012-04-17

**Authors:** David P. AuCoin, Dana E. Reed, Nicole L. Marlenee, Richard A. Bowen, Peter Thorkildson, Barbara M. Judy, Alfredo G. Torres, Thomas R. Kozel

**Affiliations:** 1 Department of Microbiology and Immunology, University of Nevada School of Medicine, Reno, Nevada, United States of America; 2 Department of Biomedical Sciences, Colorado State University, Fort Collins, Colorado, United States of America; 3 Department of Pathology, University of Texas Medical Branch, Galveston, Texas, United States of America; 4 Department of Microbiology and Immunology, University of Texas Medical Branch, Galveston, Texas, United States of America; Instituto Butantan, Brazil

## Abstract

*Burkholderia pseudomallei* is a Gram-negative bacillus that is the causative agent of melioidosis. The bacterium is inherently resistant to many antibiotics and mortality rates remain high in endemic areas. The lipopolysaccharide (LPS) and capsular polysaccharide (CPS) are two surface-associated antigens that contribute to pathogenesis. We previously developed two monoclonal antibodies (mAbs) specific to the CPS and LPS; the CPS mAb was shown to identify antigen in serum and urine from melioidosis patients. The goal of this study was to determine if passive immunization with CPS and LPS mAbs alone and in combination would protect mice from a lethal challenge with *B. pseudomallei*. Intranasal (i.n.) challenge experiments were performed with *B. pseudomallei* strains 1026b and K96423. Both mAbs provided significant protection when administered alone. A combination of mAbs was protective when low doses were administered. In addition, combination therapy provided a significant reduction in spleen colony forming units (cfu) compared to results when either the CPS or LPS mAbs were administered alone.

## Introduction

Melioidosis occurs primarily in the tropics and is caused by the soil dwelling pathogen *B. pseudomallei*. Infection with *B. pseudomallei* creates many clinical challenges, the most obvious being resistance to commonly prescribed antibiotics [1], [2], [3]. In addition, recommended treatment with effective antibiotics is intensive, consisting of a short parenteral phase followed by a long oral phase [4]. Relapse rates can approach 25%, with nearly half of these patients developing septicemia [5]. A recent prospective study determined that the incidence of melioidosis has increased in northeast Thailand from 1997–2006 and the mortality rate during this period was nearly 43% [6]. In the same geographical region, melioidosis is the third most common cause of death from infectious disease after acquired immunodeficiency syndrome (AIDS) and tuberculosis [6]. In regions of northern Australia, where intensive care treatment is more readily available, the mortality rate is still alarmingly high at 20% [2], [7].

*B. pseudomallei* encodes many well-established virulence factors, two of which are the capsular polysaccharide (CPS) and lipopolysaccharide (LPS) [8], [9], [10], [11], [12], [13], [14]. CPS is an unbranched homopolymer of 1,3-linked 2-*O*-acetyl-6-deoxy-β-D-*manno*-heptopyranose residues. [15]. A subtractive hybridization study determined that the CPS is a major virulence factor necessary for pathogenicity in a Syrian hamster model of acute melioidosis [8]. In the same study, a CPS mutant strain was 10,000-fold less virulent when compared to a wild type strain. CPS also reduces the amount of complement factor C3b deposited on the bacterial surface, which in turn confers resistance to phagocytosis [9].

*B. pseudomallei* LPS contributes to pathogenesis *in vitro* and *in vivo*. The O-antigen component of LPS is an unbranched polymer of 1,3 linked β-d-glucopyranose-(1–3)-6-deoxy-α-l-talopyranose residues [15], [16]. A *B. pseudomallei* O-antigen mutant is more vulnerable to killing by a mouse macrophage cell line [11] and more susceptible to killing through the alternative complement pathway [12], [13]. In human melioidosis cases, survivors develop an IgG3 antibody response specific to LPS [10], [14].

The goal of this study was to evaluate the therapeutic potential of two mAbs specific to the LPS and the manno-heptose CPS of *B. pseudomallei* [17]. Our study (i) challenged mice with two strains of *B. pseudomallei* via the i.n. route, (ii) administered mAbs alone and in combination, and (iii) assessed survival, spleen colony forming units (cfu), and organ abscess formation. The data generated supports and strengthens previous findings that indicate targeting *B. pseudomallei* surface expressed polysaccharides for treatment of melioidosis may be a sensible endeavor.

## Materials and Methods

### Immunization of mice and production of mAbs

Production of IgG3 mAbs 4C7 (LPS) and 3C5 (CPS) has been previously described [17]. Briefly, *B. pseudomallei* strain 1026b was grown overnight at 37°C in brain heart infusion media under BSL-3 containment practices. BALB/c mice were then immunized with 2×10^8^ heat-inactivated *B. pseudomallei* (80°C for 2.5 h) by the intraperitoneal (i.p.) route every two weeks for an eight-week period [18]. An enzyme-linked immunosorbent assay, with heat-inactivated strain 1026b in the solid phase, was used to assess antibody titers to *B. pseudomallei* [18]. The last immunization was administered three days prior to harvest of spleens. Hybridoma cells were produced as previously described [19]. Western blot analysis was done to identify hybridoma cell lines that were producing mAbs reactive with purified CPS or producing a ladder pattern characteristic of LPS binding. Hybridoma cell lines were grown in Integra CL 1000 culture flasks (Integra Biosciences) and mAbs were purified by affinity chromatography over a protein-A column.

### Immunohistochemistry (IHC)

mAbs 3C5 and 4C7 were coupled to horseradish peroxidase (HRP) using the EZ-Link Plus™ Activated Peroxidase Kit (Pierce) followed by purification with a Pierce Conjugate Purification Kit. Tissue sections were deparaffinized using Histoclear (National Diagnostics) followed by a graded ethanol series. Hydrogen peroxide (0.3%) was applied to tissues to reduce endogenous peroxidase activity. Sections were incubated in 0.15 M glycine in PBS for 15 min, rinsed in PBS, and then incubated in a blocking solution (1% BSA/PBS) for 30 min. HRP-labeled mAbs were diluted to 15 µg/ml in blocking solution and applied to the tissues for 3 h at room temp. Slides were rinsed with PBS followed by treatment with diaminobenzidine (DAB) substrate solution (ImmPACT™ DAB Peroxidase Substrate, Vector Laboratories). Slides were washed in water and counterstained using hematoxylin. Microscopy was performed using a Nikon Eclipse E800 microscope and a Spot RT color digital camera (Diagnostic Instruments).

### Intranasal challenge model

Two murine melioidosis infection models were used under ABSL-3 containment practices. The first model began by injecting female BALB/c mice with one dose of various concentrations of mAb(s) (3C5, 4C7, or IgG3 subclass control mAb) by the i.p. route, 18 h prior to challenge. A vial of frozen *B. pseudomallei* 1026b was thawed and diluted in PBS to a concentration of approximately 5000 cfu/25 µl (15 LD_50_). Mice were anesthetized, held vertically, and 25 µl of the inoculum was released into the nares for inhalation. Following challenge, the inoculum was back titrated on agar plates to confirm delivered dose. Mice were weighed prior to inoculation, daily for 10 days, then twice weekly until 3 or 6 weeks post-challenge. Using this model, control mice became debilitated and required euthanasia 3–4 days post-challenge. At necropsy, the internal organs were excised aseptically and examined by one of two veterinarians for the presence of abscesses (the number and size of each abscess were noted). Spleens were then homogenized in 1 ml of LB broth using a mixer mill. The homogenate (100 µl) was plated on LB plates and colonies counted 2 days later to determine bacterial loads.

The second i.n. challenge model was modified from a previously described protocol [20]. Briefly, female BALB/c mice were administered various doses of mAb via the i.p. route 18 h prior to infection with *B. pseudomallei* strain K96423. Mice were then challenged via the i.n. route (50 µl) with approximately 600 cfu (2 LD_50_). Mice were weighed prior to inoculation and monitored for 21 days post-infection. Using this model, control mice became debilitated and required euthanasia 4–6 days post-challenge. For all passive immunization experiments, control mice were untreated or were administered an isotype control IgG3 mAb (F26G3) specific to the capsule of *Bacillus anthracis* [19], [21].

### Statistics

All statistical analysis was performed with Prism 5 software (GraphPad Software, Inc.) Survival curves were generated by use of Kaplan-Meier estimators. The survival distributions of each treatment group vs. control mice were compared via the log-rank (Mantel-Cox) test. Significance of spleen cfu vs. control mice were calculated with Fisher's exact test.

### Ethics Statement

This study was carried out in accordance with the recommendations in the Guide for the Care and Use of Laboratory Animals of the National Institutes of Health. The protocols were approved by the Animal Care and Use Committees of Colorado State University (Protocol #09-001A) and the University of Texas Medical Branch (Protocol #0503014A). Mice were anesthetized by intraperitoneal injection of a ketamine/xylazine solution.

## Results

Our previous study determined that IgG3 mAbs 4C7 and 3C5 are reactive with *B. pseudomallei* LPS and CPS, respectively [17]. By Western blot mAb 4C7 produces a ladder pattern typical of *B. pseudomallei* LPS binding [22], [23], [24], [25] and mAb 3C5 is reactive with purified CPS that was structurally verified by nuclear magnetic resonance (NMR) [17]. Before proceeding with passive immunization studies we confirmed by immunofluorescence that mAbs 3C5 and 4C7 are reactive with the exterior of *B. pseudomallei* cells (data not shown).

**Figure 1:**
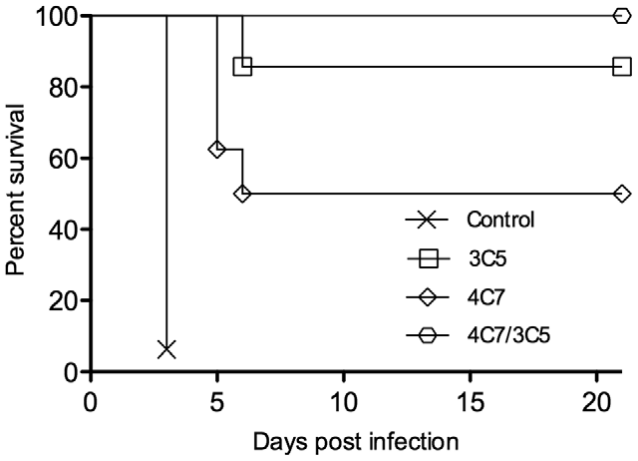
Protection in passively immunized mice following i.n. challenge with *B. pseudomallei* strain 1026b. BALB/c mice were administered 1 mg of either CPS IgG3 mAb 3C5 or LPS IgG3 mAb 4C7 alone or 1 mg of each mAb in combination by the i.p. route. Intranasal challenge was performed 18 h later with 15 LD_50_ of *B. pseudomallei*. Mice were monitored for 21 days after which gross pathology and spleen cfu were determined on survivors (Table 1). Control mice were treated with 1 mg of an irrelevant IgG3 mAb. *p* values of survival vs. controls are listed in Table 1.

The mAbs were tested for the ability to provide passive protection in a murine model of pulmonary melioidosis. An initial study determined that i.n. challenge with 5000 cfu (equivalent to 15 LD_50_) of *B. pseudomallei* strain 1026b caused development of acute disease in BALB/c mice (data not shown). At this challenge dose, mice were euthanized within 2–3 days. The initial passive immunization study consisted of i.p. administration of 1 mg of mAb 3C5 or 4C7 alone and 1 mg of each mAb in combination; these doses did not cause any adverse effects in the mice. Mice were challenged 18 h post mAb treatment with an estimated dose of 5000 cfu of *B. pseudomallei* and monitored for 21 days. At this dose all infected mice showed reduced activity and had ruffled fur. Administration of mAb 3C5 protected 86% (6/7) of the infected mice and mAb 4C7 protected 50% (4/8) (Fig. 1; Table 1, Exp. 1). In addition, all of the mice that received both mAbs in combination (1 mg of each mAb/mouse) survived the challenge. All of the mice from this initial study lost weight 1–2 days following challenge, however, mice that survived began to gain weight back by day 6 and appeared healthy by day 21. Control mice were injected with 1 mg of an IgG3 mAb specific for the capsule of *B. anthracis* [19], [21]; these mice became moribund and were euthanized at day 3.

**Table 1:** Survival and gross pathology of mice passively treated with mAbs.

mAb dose (µg)			Study length (days)	Survived (*p* value)^a^	(+) Spleen cfu^b^ (*p* value)^c^	Spleen cfu of survivors^d^	Abscess formation^e^
CPS (3C5)	LPS (4C7)	IgG3 control					
Experiment 1 - *B. pseudomallei* strain 1026b							
-	-	1000	21	**0/8**	-	-	**-**
1000	-	-	21	**6/7 (<0.01)**	5/7 ( = 0.20)	0,0,1,2,106,T	0/6
-	1000	-	21	**4/8 (<0.01)**	7/8 ( = 0.50)	0,12,T,T	3/4
1000	1000	-	21	**6/6 (<0.01)**	**0/6 (<0.01)**	0,0,0,0,0,0	0/6
Experiment 2 - *B. pseudomallei* strain 1026b							
-	-	-	42	0/8	-	-	-
1000	-	-	42	**6/8 (<0.01)**	5/8 ( = 0.10)	0,0,0,214,T,T	1/6
500	-	-	42	**4/8 (<0.01)**	5/8 ( = 0.10)	0,0,0T	1/4
250	-	-	42	0/8 ( = 0.48)	8/8 (>0.50)	-	-
125	-	-	42	**7/8 (<0.01)**	5/8 ( = 0.10)	0,0,0,4,5,T,T	3/7
-	2000	-	42	0/8 (<0.20)	8/8 (>0.50)	-	-
-	1000	-	42	**4/8 ( = 0.01)**	7/8 ( = 0.50)	0,T,T,T	0/4
-	500	-	42	1/8 ( = 0.06)	7/8 ( = 0.50)	0	0/1
-	250	-	42	**0/8 ( = 0.01)**	8/8 (>0.50)	-	-
Experiment 3 – *B. pseudomallei* strain 1026b							
-	-	-	42	0/8	-	-	-
500	1000	-	42	**5/8 (<0.01)**	7/8 ( = 0.50)	0,2,6,T,T	2/5
250	500	-	42	**7/8 (<0.01)**	**2/8 (<0.01)**	0,0,0,0,0,0,1	0/7
125	250	-	42	**7/8 (<0.01)**	**2/8 (<0.01)**	0,0,0,0,0,0,T	1/7
62.5	125	-	42	**7/8 (<0.01)**	**4/8 ( = 0.04)**	0,0,0,0,128,T,T	2/7
Experiment 4 – *B. pseudomallei* strain K96423							
-	-	1000	21	0/8	-	-	-
1000	-	-	21	**6/8 (<0.01)**	-	-	-
250	-	-	21	**3/8 (<0.01)**	-	-	-
62.5	-	-	21	**4/8 (<0.01)**	-	-	-
16	-	-	21	**2/8 (<0.01)**	-	-	-
-	1000	-	21	**2/8 (<0.01)**	-	-	-
-	250	-	21	**3/8 (<0.01)**	-	-	-
-	62.5	-	21	**1/8 (<0.01)**	-	-	-
-	16	-	21	**2/8 ( = 0.02)**	-	-	-

a *p* value vs. controls determined from Kaplan-Meier survival plots by log-rank (Mantel-Cox) test, bold values are statistically significant (*p*<0.05).

b positive spleen cfu was determined on survivors and assumed to occur in mice that died before study endpoint.

c *p* values vs. controls determined by Fisher's exact test, bold values are statistically significant (*p*<0.05).

d spleen cfu was assessed on survivors only; values indicate cfu determined by plating 100 µl from a 1 ml spleen homogenate; T indicates too numerous to count.

e determination of abscess formation on internal organs was performed on survivors only.

In addition to survival, the effect the mAbs had on development of spleen cfu and abscess formation was determined. Spleen cultures were determined for survivors only. Spleen culture data is included for each treatment group in Table 1. Additionally, mice that did not survive the challenge dose were assumed to develop spleen cfu in order to allow for a statistical evaluation of spleen cfu in treatment groups vs. controls (Table 1). Spleen cfu developed in 71% (5/7) and 88% (7/8) of mice treated with mAbs 3C5 and 4C7, respectively (Table 1; Exp. 1). A statistically significant reduction in spleen cfu occurred when both mAbs were administered in combination; all six of the mice in this group did not develop spleen cfu. Abscess formation developed in 75% (3/4) of the surviving mice that were administered mAb 4C7, whereas mice administered mAb 3C5 or both mAbs in combination did not develop abscesses.

**Figure 2:**
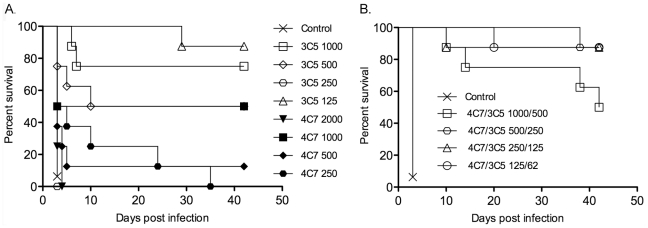
Effect of mAb dose and combination therapy in mice challenged with *B. pseudomallei* strain 1026b. Mice were administered mAb(s) by the i.p. route followed 18 h later by i.n. challenge with 15 LD_50_ of *B. pseudomallei*. (A) Dose-response experiment in which mice were treated with the doses (µg) listed of each mAb alone. (B) Multiple doses of mAbs 3C5 and 4C7 were administered in combination at the doses (µg) listed. Mice were monitored for 42 days after which gross pathology and spleen cfu were determined on survivors (Table 1). Control mice were not treated with mAb. *p* values of survival vs. controls are listed in Table 1.

**Figure 3:**
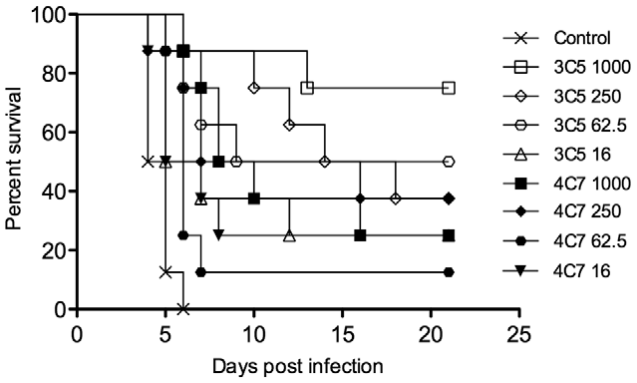
Protection in passively immunized mice following i.n. challenge with *B. pseudomallei* strain K96243. mAbs were administered by the i.p. route at the doses (µg) listed. Intranasal challenge was performed 18 h later with 2 LD_50_ of *B. pseudomallei*. Mice were monitored for 21 days. Control mice were treated with 1 mg of an irrelevant IgG3 mAb. *p* values of survival vs. controls are listed in Table 1.

A dose-response experiment was performed to estimate the median effective dose (ED_50_) of each mAb (Fig. 2A; Table 1, Exp. 2). Experiment 1 suggested that mAb 4C7 was not as protective as mAb 3C5. Therefore, a higher starting dose of mAb 4C7 was used in the dose response experiment compared to mAb 3C5. In addition mice were monitored for 42 days to determine if survival rates and gross pathology would be adversely affected. Three of the four doses of mAb 3C5 provided highly significant protection with the 125 µg dose providing optimal protection (88% survival). The 250 µg dose of mAb 3C5 did not protect any of the mice. This result was most likely due to experimental error since the doses higher and lower than 250 µg were protective, and this lack of protection was not observed in a second experiment with the K96243 strain (Fig. 3). At the highest dose of mAb 4C7 (2000 µg) no protection was achieved, however, at 1000 µg half of the mice survived (4/8) (similar to Exp. 1). Lower doses of mAb 4C7 did not protect as many mice as mAb 3C5. None of the doses of either mAb produced a statistically significant reduction in spleen cfu, similar to the individual doses in Exp. 1.

Complete protection with no indication of pathology was achieved when 1 mg of each mAb was administered in combination. Therefore, an additional combination experiment with multiple doses was performed (Fig. 2B; Table 1, Exp. 3) in order to access synergy [26], [27]. In designing the synergy experiment the highest doses were estimated ED_50_ values of each mAb based on the results from Fig. 2A. Therefore, the highest dose administered was 500 µg of mAb 3C5 combined with 1000 µg of mAb 4C7. Three smaller doses (twofold serial dilution from the highest doses) were also administered and mice were monitored for 42 days. All doses provided significant protection; the lowest three doses each protected 7/8 mice. In addition, the three lowest doses of combination therapy significantly reduced spleen cfu, a reduction not seen in any of the individual mAb dose experiments.

An additional dose-response experiment was performed to analyze protection against a different strain of *B. pseudomallei* (K96243). mAb 3C5 protected 7/8 mice at the 125 µg dose (Table 1; Exp. 2), so a four-fold dilution of mAb doses were used (from 1000 µg to 16 µg) in this experiment to determine potency at lower doses. Mice were infected via the i.n. route with 2 LD_50_ of *B. pseudomallei* strain K96243 and monitored for 21 days. All of the doses provided statistically significant protection compared to control mice. In accordance with the previous experiments, CPS mAb 3C5 appeared to be more potent than LPS mAb 4C7 at identical doses; however, this difference was not statistically significant.

**Figure 4:**
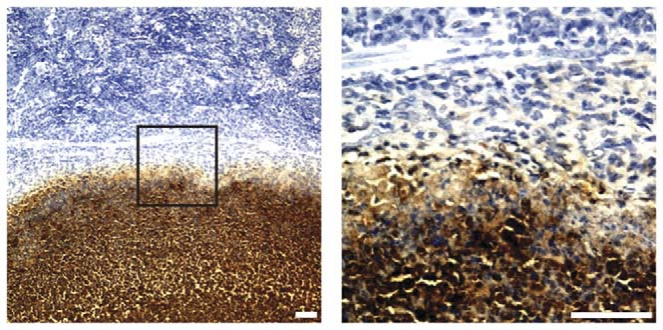
Detection of CPS within a splenic abscess by IHC. Organs were harvested from control BALB/c mice (Fig. 3) that were infected with *B. pseudomallei* strain 1026b. A tissue section from a spleen that contained multiple large abscesses is shown (left panel). Location of CPS was identified by HRP-labeled mAb 3C5 (brown). Box within the panel on the left indicates the boundary of an abscess and surrounding normal splenic tissue (tissue within box is magnified in right panel). White scale bars indicate 50 µm.

Abscess formation occurred in 20% (13/64) of surviving mice (Exp. 1–3) with the most common site being spleen (67%) followed by liver (20%) and lung (13%) (data not shown). The ability of CPS mAb 3C5 and LPS mAb 4C7 to detect antigen within an abscess by IHA was determined. Spleen tissue sections were used since the percentage of abscess formation in this organ was highest. Spleens were harvested from control mice that did not receive mAb treatment. Fig. 4 displays an IHC image of the outer edge of an abscess where CPS was identified with HRP-labeled mAb 3C5 (brown) surrounded by splenic tissue (blue). mAb 4C7 was unable to detect LPS within a section of the same splenic abscess. It is unclear whether this is due to (i) alteration of the LPS structure during preparation of the tissue for IHC (ii) the CPS antigen being more abundant on the bacterial cell surface or (iii) the CPS being more accessible to antibody when contained within an abscess.

## Discussion

A number of successful passive immunization studies have been performed that administered either polyclonal (pAb) [28], [29], [30] or monoclonal antibodies [18], [29], [31], [32] reactive with polysaccharides in an effort to understand the pathogenesis of *B. pseudomallei*. These studies administered antibodies reactive with LPS and/or uncharacterized high molecular weight capsular polysaccharides. There are four known capsular structures in *B. pseudomallei* [33], which complicates the identification of which specific capsular polysaccharide was targeted in the previous studies. Therefore, one goal of the current research was to characterize the protection afforded by CPS mAb 3C5, which has been previously shown to bind to purified (NMR verified) 2-*O*-acetyl-6-deoxy-β-D-*manno*-heptopyranose capsule [17].

The current report provides additional support for the development of vaccines and therapeutic antibodies targeting surface exposed polysaccharides of *B. pseudomallei*. The data demonstrate that LPS mAb 4C7 is able to provide significant passive protection against two different strains of *B. pseudomallei*. This is consistent with previous passive immunization studies that targeted *B. pseudomallei* LPS [18], [28], [29], [30], [31]. Interestingly, the 2000 µg dose of mAb 4C7 did not protect any of the mice, while the 1000 µg dose protected half of the mice (Table 1, Exp. 1 & 2). This may be due to a prozone phenomenon, in that high doses of polysaccharide-specific mAbs have been shown to inhibit protective effects [34], [35], [36]. mAb 3C5, which is specific to the manno-heptose capsule of *B. pseudomallei,* also provided significant passive protection. In addition, mAb 3C5 was able to protect more mice at lower doses compared to mAb 4C7, although this was not statistically significant. A number of studies have successfully targeted unknown capsular polysaccharides with passive mAb therapy [18], [31], [32]. Our study appears to be the first to provide passive protection with a mAb reactive to a specific capsular polysaccharide.

The mAbs in this study were individually protective; therefore we anticipated that the mAbs would be more effective if administered in combination. An informative study by Jones et al. determined that administration of a combination of LPS, unknown protein, and high molecular weight polysaccharide specific antibodies was able to protect against an i.p. challenge with *B. pseudomallei* [18]. When CPS mAb 3C5 and LPS mAb 4C7 were administered in combination the three lowest dose combinations were able to protect 88% of the mice (21/24). This high level of protection suggested synergistic effects between the two mAbs. A statistical assessment of synergy [26], [27] was performed with CalcuSyn software, however, values could not be calculated due to the high levels of protection at the lowest combination doses. The result adds to the previous study by Jones et al. by achieving protection from i.n. challenge with combination therapy consisting of mAbs specific to LPS and the manno-heptose capsule.

At all doses, bacterial colonization of the spleen was not effectively controlled when mAbs were administered alone. Combination therapy at the lower doses resulted in a significant reduction in the development of spleen cfu and low numbers of abscesses in survivors. The two intermediate combination doses summarized in Table 1 (Exp. 3) illustrate this point well. Of the 14 of 16 mice that survived, only two developed spleen cfu and one developed an abscess. As mentioned, one previous study found combination therapy to be effective against a *B. pseudomallei* i.p. challenge [18]. However, most of the surviving mice developed abscesses on the spleen and liver [18]. Our data suggest a benefit of low dose combination therapy based on the significant reduction in spleen cfu and low levels of abscesses on internal organs of survivors. It is not clear whether one of the mAbs is more effective at controlling spleen cfu and abscess formation, however it is interesting that only mAb 3C5 (and not mAb 4C7) was able to identify CPS present in abscesses by IHC (Fig. 4).

There is no effective vaccine available to prevent melioidosis and treatment of the disease remains challenging. Although many vaccination studies in animals have been completed, none have elicited sterilizing immunity [37]. Antibiotic treatment in humans is also difficult; even with the administration of appropriate antibiotics, relapse rates remain high [5], [38]. Therefore, adjuncts to antibiotic therapy are greatly needed. Several studies have been undertaken to identify effective adjunctive treatments. One study administered low-dose hydrocortisone, along with ceftazidime, for the treatment of severe sepsis in mice, although this did not provide a survival benefit [39]. Cheng et al. concluded that adjunct treatment with granulocyte colony-stimulating factor (G-CSF) might have contributed to reduction in mortality among melioidosis patients with septic shock [40]. However, the benefit of G-CSF treatment was not supported in a murine model of melioidosis or an *in vitro* whole blood bactericidal assay [41], [42]. Finally, an encouraging study by Propst et al. concluded that administration of gamma interferon improves survival in a murine model of pulmonary melioidosis [43].

The current study has determined that *B. pseudomallei* polysaccharide specific mAbs can provide significant protection in a murine model of pulmonary melioidosis when administered alone. Significant protection was also achieved when both mAbs were administered in combination. In addition, development of spleen cfu was significantly reduced when mAbs were administered in combination as compared to mAbs administered alone. Administration of both protective antibodies appeared to elicit synergistic, or at the very least additive, effects in our studies. The combination therapy was an attempt to mimic the natural polyclonal response to infection. Therefore, the mAbs described in this report may have potential as an adjunct therapy to antibiotics. Future studies will evaluate the benefit of administration of relevant antibiotics along with CPS and LPS mAbs (alone and in combination). If mAb adjunct therapy is effective, additional studies will evaluate the treatment in a post-challenge model. Such a treatment may aid in reducing the development of latent foci that would eventually cause relapse of disease.
